# Postpartum Use of Shavari Bar® Improves Breast Milk Output: A Double-Blind, Prospective, Randomized, Controlled Clinical Study

**DOI:** 10.7759/cureus.26831

**Published:** 2022-07-13

**Authors:** Amita Birla, Meena Satia, Rita Shah, Arnav Pai, Shruti Srivastava, Deepak Langade

**Affiliations:** 1 Clinical Research, Act Lifesciences Pvt Ltd., Navi Mumbai, IND; 2 Obstetrics and Gynecology, Dr. DY Patil Hospital and Research Centre, Navi Mumbai, IND; 3 Prenatal and Postnatal Care, NM Medical Center, Mumbai, IND; 4 Gynecology, Dr. DY Patil Hospital and Research Centre, Navi Mumbai, IND; 5 Pharmacology, Dr. DY Patil University, School of Medicine, Navi Mumbai, IND

**Keywords:** asparagus racemosus, shavari bar, post-partum nutrition, breast-feeding, lactation, shatavari

## Abstract

Background and objectives

Appropriate nutrition, along with the establishment of lactation, is of paramount importance for the feeding mother and the growing neonate. *Asparagus racemosus*, a common name for Shatavari, is a well-known herb that has been used as a galactagogue in traditional Indian culture. It is also referenced in Ayurvedic medicine. Despite multiple formulations available, palatability has been a concern always as Shatavari is very bitter. We have devised a palatable and nutritionally rich formulation of Shatavari with no artificial ingredients. To understand the efficacy, we have conducted this double-blind, prospective, randomized, controlled study to evaluate the effect of oral Shatavari formulation (Shavari Bar®) on breast milk output in postpartum women.

Methods

A prospective, randomized, parallel-group, double-blind, placebo-controlled study was conducted at two centers in women with gestational age 37 weeks or more who intended to breastfeed. Hundred and four women were screened, of which 78 were randomized to receive either bar containing Shatavari and oats (n=39, study) or an identical placebo bar (n=39, control). All 78 women completed the study, 61 delivered by a lower segment Caesarean section (LSCS), and 17 had a full-term normal vaginal delivery. Time to first noticeable breast fullness was measured and expressed milk volume measurements were done 72 hours after delivery or after consumption of four bars, whichever was later using a standardized breast pump. Comparison between the two groups was analyzed using a t-test.

Results

Demography and baseline data of patients enrolled were similar in the two groups. The mean total milk volume expressed was higher (p=0.008) with Shavari (64.74 ml) compared to placebo (49.69 ml). The time to breast fullness was shorter (p=0.024) with Shavari (30.49 hours) compared to placebo (38.09 hours). No adverse events were noted in either of the study groups. Global assessment of the satisfaction of mothers with lactation, the well-being of the child, taste, and ease of use was better in the treatment arm than in the placebo arm.

Conclusion

The use of the Shavari bar can be an effective option in postpartum women to establish early lactation and build confidence in breastfeeding along with nonpharmacological intervention.

## Introduction

Breast milk provides the ideal nutrition for the infant, and World Health Organization (WHO) recommends exclusive breastfeeding for the initial six months. Breastfeeding is one of the most effective ways to ensure child health and survival. Adequate milk production is not only critical, but the initial early milk production has been shown to significantly affect milk production during the established lactation phase. However, nearly two out of three infants are not exclusively breastfed for the recommended six months, a rate that has surprisingly not improved in two decades despite multiple educational initiatives by the healthcare professional (HCP) community [1]. Many women express concern about their ability to produce enough milk, and insufficient milk production has been cited as a reason for supplementation and early cessation of breastfeeding [2].

A longitudinal observational study that enrolled mothers who had initiated breastfeeding after delivering healthy-term infants found that the rate of discontinuation of breastfeeding was nearly 37% by week two [3]. There was a high frequency of concerns about inadequate milk production in early lactation which led to the introduction of water or top feeds in the initial postpartum days. If supplementary feeds were given instead of breastfeeds, they might have a negative impact on milk supply. Adequate milk supply in the first few weeks postpartum is critical, and nonpharmacological interventions are commonly practiced, like skin-to-skin contact, breastfeeding within an hour of birth, and frequent breastfeeding during the first 24 hours after birth [4]. Oral galactagogues, substances that stimulate milk production, may be used to improve breast milk output.

Shatavari (*Asparagus racemosus*), also known as "wild asparagus", is a plant native to the Indian subcontinent and used in Ayurvedic medicine. It has a long history of use as a galactagogue in India and is also included in the official ayurvedic pharmacopeia for this use [5]. The primary active constituents of *A. racemosus* are steroidal saponins found in the roots [5]. It is loaded with folic acid, vitamins A, C, and K, and phytoestrogens; the hormonal effect of phytoestrogens is like estrogen in milk production. A key regulator of prolactin production is estrogens which enhance the growth of prolactin-producing cells and stimulate prolactin production directly, as well as by suppressing dopamine. It also contains tryptophan, an essential amino acid that may stimulate prolactin production, leading to increased milk production [6].

Shatavari is available in various forms like powder, granules, capsules, etc. However, many of these formulations use a very high sugar content to mask the bitter taste of Shatavari. Also, most of these products must be mixed with milk, so the palatability and taste are of concern. We have developed a unique formulation of Shatavari, Shavari Bar®, which is a granola bar having Shatavari and oats along with dry fruits, honey, and sweetened cocoa. It is natural and preservative-free. This study was conducted to evaluate the product's efficacy and safety, and palatability in the postpartum phase for breastfeeding mothers.

## Materials and methods

Objectives

This study evaluated the efficacy and safety of oral administration of Shavari Bar® versus placebo bar in increasing breast milk output during the postpartum period.

Study design and setting

The prospective, randomized, parallel-group, double-blind, placebo-controlled study was conducted at two obstetric settings, DY Patil Hospital in Navi Mumbai and NM Medical Center in Mumbai. Following the international mandate, the study protocol was designed per the Declaration of Helsinki developed by the World Medical Association (2013 amendment). The Institutional Ethics Committee approved the study protocol and documents, DY Patil Medical College and Hospital, Navi Mumbai (No. DYP/EC/15/2021; July 9, 2021) and registered with the clinical trials registry of India (CTRI/2021/08/035753; August 18, 2021). The study was conducted in accordance with the Good Clinical Practice (GCP) guidelines and informed written consent was obtained from all participants before starting any study-related procedures. The study was conducted between August 2021 and May 2022. The entire study was conducted and reported following the Consolidated Standard of Reporting Trials (CONSORT) statement.

Study participants

Pregnant women with the gestational age of 37 weeks or more who intend to breastfeed were invited to participate in this study. Women who delivered by vaginal delivery or lower segment Cesarean section (LSCS) delivery were enrolled.

Inclusion Criteria

Healthy women between 20-40 years of age who signed informed consent, with uncomplicated full-term delivery (vaginal or LSCS), women who have accomplished antenatal breastfeeding promotion protocol immediately postpartum or within three days of delivery, and women able to understand the study requirements and can fill the study log diary, and follow other procedures required by the study protocol were enrolled.

Exclusion Criteria

Postpartum women with contraindications to breastfeeding, such as HIV, chemotherapeutic drugs, radioactive substances, and babies with galactosemia, were excluded. Postpartum women with unstable conditions (i.e., postpartum hemorrhage, sepsis). Women with known allergies to Shatavari or oats, raisins, almonds, cocoa, and honey were excluded. Women whose babies require phototherapy, women with insufficient glandular tissue or breast surgery and any structural abnormality of the breast were excluded. Women with a history of infertility, hypothyroidism, women with twins, or higher-order births were excluded. Any known clinically significant endocrine, metabolic, hepatic, renal, cardiovascular, gastrointestinal, respiratory, hematological, or neurological illnesses or the presence of any current psychiatric disorders in women were considered as exclusion criteria. If any other investigational drug was used within three months before the entry in this study or those who cannot be relied upon to comply with the test procedures or are unwilling to give informed consent were excluded from the study.

Sample size, randomization, and blinding

It was planned to enroll 80 subjects in this exploratory study with a randomization ratio of 1:1. Randomization was done using a computer-based pre-determined randomization program (Rando Version 1.0) in a block of 20. Thus, there were four blocks of 20 in each, with each block containing 10 patients in the study group and 10 in the control group. Both patients and study group members, who conducted and assessed the outcomes, were unaware of the treatment received by the women. Blinding was done by preparation of a placebo bar that was identical in size, shape, color, and taste to the Shavari bar. The packaging was labeled to conceal the contents of the packet. Women who qualified for the study during screening were allocated a serial number in a sequence, and the women received the treatment packet based on the serial number (study number) allocated to the patient. The randomization codes were maintained in separate sealed envelopes accessible only to the principal investigator in an emergency.

Study outcomes

The primary outcome was the total volume (ml) of breast milk produced on the third postpartum day (72 hours after delivery) or after taking four doses of the study medication, whichever is later, during 15 minutes of pumping both breasts using a breast pump/manually two hours after breastfeeding.

Secondary efficacy outcomes were: i) time to noticeable breast fullness after delivery; ii) subjective satisfaction of mother regarding the well-being and happiness of newborn; iii) subjective satisfaction of mothers regarding the state of lactation; iv) subjective satisfaction of investigator regarding the well-being of the mother, and v) subjective satisfaction of investigator regarding the well-being of the newborn. Secondary safety outcomes were: i) proportion of patients experiencing treatment-emergent adverse events (TEAEs), and ii) subjective satisfaction of patients with the taste and ease of use of the product. All subjective assessments were assessed on a five-point Likert scale (very satisfied, satisfied, neutral, unsatisfied, very unsatisfied).

Safety was assessed based on the spontaneous reports generated by the patients/clinicians. All adverse events (AEs) and serious adverse events (SAEs) were reported as per local regulatory guidelines.

Interventions

Control Group (Placebo): Group A

The control group received a placebo formulation (bar containing oats, dry fruits, honey, and chocolate-flavored) not containing Shatavari. The placebo bar was identical in size, shape, and appearance to the study formulation (Shavari Bar®). Women continued their routine postpartum care as per the hospital protocol.

Study Group: Group B

**Figure 1:**
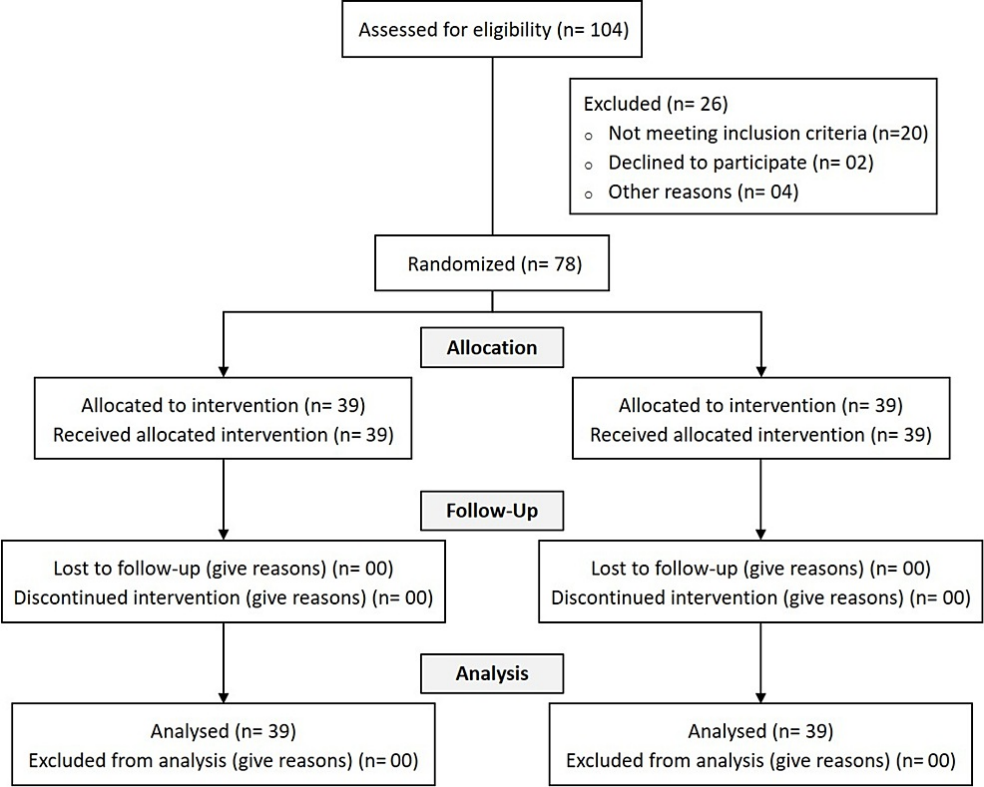
CONSORT flowchart CONSORT - Consolidated Standard of Reporting Trials

The study group received oral supplementation with the study product (Shavari Bar®) for five days, starting on day two after delivery (the first postpartum day). Shavari Bar® contained Shatavari, oats, dry fruits, and honey and was chocolate flavored. Women continued their routine postpartum care as per the hospital protocol. Figure 1 presents the CONSORT flowchart for patients in the study.

Study procedures

Informed Consent

Informed consent was obtained before starting any study-related procedures on the patients. 

Clinical and Physical Examinations

All participants underwent a complete clinical examination on enrolment to rule out any abnormalities. The medical history of all the women was taken before study initiation. Vital parameters were reported (pulse, blood pressure, temperature, and respiratory rate). Women underwent general and systemic examinations (cardiovascular, respiratory, abdominal, nervous, and musculoskeletal systems).

Time to Noticeable Breast Fullness

Time to evident breast fullness is defined as the mean time from birth to evident breast fullness. The participants were asked if they noticed their breasts were full, followed by the question: "When did you feel breast fullness?".

Milk Volume Measurements

Milk volume was measured after the breast milk expression from both breasts using a breast pump. An electric breast pump with three-phase pumping, which includes massage, stimulation, and expression, was used. Manual extraction was done in case of failure to use a breast pump.

Subjective Satisfaction

Subjective assessment for satisfaction by mothers regarding the well-being and happiness of babies and the state of lactation were assessed on a five-point Likert scale: very satisfied, satisfied, neutral (neither satisfied nor dissatisfied), unsatisfied, very unsatisfied. Subjective satisfaction of the investigator was assessed on a five-point Likert scale, as noted above. Subjective satisfaction of patients on the taste and ease of use of the product was on a five-point Likert scale, as noted above.

Compliance

Women were asked to maintain a log for study medication for medication compliance.

Safety Assessment

Clinical safety was assessed by evaluating adverse events reported and/or observed during the study. The patients reported adverse events during the follow-up or the clinical evaluation of patients.

## Results

The complete set of data was analyzed, and also LSCS and normal delivery subgroup analysis was carried out. In the total study population, 61 patients had undergone LSCS, and 17 were normal delivery patients. The demographic baseline data of both arm A and arm B were comparable in terms of age, height, weight, and body mass index (BMI). Vitals like pulse, blood pressure, and temperature are presented in Table 1.

**Table 1:** Demography and baseline data of patients enrolled BMI - body mass index; DBP - diastolic blood pressure; FTND - full-term normal delivery; LSCS - lower segment Caesarean section; SBP - systolic blood pressure

Data	Control group (placebo)			Study group (Shavari Bar®)			t-test
	N	Mean	SD	N	Mean	SD	p
LSCS delivery (n=61)							
Age (years)	31	29.19	5.69	30	28.93	3.22	0.827
BMI (kg/m^2^)	31	28.78	3.91	30	29.19	4.28	0.698
SBP (mm Hg)	31	118.32	8.53	30	116.87	8.03	0.496
DBP (mm Hg)	31	75.87	5.11	30	74.93	4.81	0.464
Pulse rate (per min.)	31	81.81	8.72	30	82.07	8.95	0.909
Temperature (F)	31	96.65	1.32	30	96.79	1.31	0.750
Time after starting drug (hours)	31	70.03	1.92	30	71	2.29	0.079
FTND (n=17)							
Age (years)	8	28.5	2.62	9	27.44	3.47	0.494
Height (cm)	8	155	4.24	9	147.67	24.67	0.421
Weight (kg)	8	71.38	17.56	9	74.4	11.56	0.677
BMI (kg/m^2^)	8	29.63	6.67	9	37.32	16.21	0.231
SBP (mm Hg)	8	115.75	4.83	9	116.67	6.16	0.740
DBP (mm Hg)	8	77	4.41	9	75.78	5.14	0.609
Pulse rate (per min.)	8	85.25	6.58	9	74.22	25.39	0.253
Temperature (F)	8	97.3	0.79	9	97.53	0.12	0.636
Time after starting drug (hours)	8	70.75	2.05	9	70.78	2.73	0.982
All patients (n=78)							
Age (years)	39	29.05	5.19	39	28.59	3.29	0.640
Height (cm)	39	157.54	8.43	39	153.03	12.72	0.069
Weight (kg)	39	71.74	11.82	39	70.93	11.5	0.758
BMI (kg/m^2^)	39	28.95	4.51	39	31.06	9.02	0.195
SBP (mm Hg)	39	117.79	7.93	39	116.82	7.56	0.580
DBP (mm Hg)	39	76.1	4.94	39	75.13	4.83	0.381
Pulse rate (per minute)	39	82.51	8.37	39	80.26	14.42	0.401
Temperature (F)	28	96.83	1.22	17	96.92	1.21	0.808
Time after starting drug (hours)	39	70.18	1.94	39	70.95	2.36	0.121

There was a significant increase in total volume (ml) of breast milk produced on the third postpartum day (72 hours after delivery) or taking four doses of the study medication, whichever is later, measured after breast milk expression from both breasts using a breast pump. An electric breast pump with three-phase pumping, which includes massage, stimulation, and expression, was used for 15 minutes of pumping both breasts two hours after breastfeeding. Similarly, the time to breast fullness was much lesser in the treatment arm as compared to the placebo arm. The time to breast fullness was 38.09 hours and 30.49 hours, respectively, in the placebo vs. treatment arm, and milk volumes were 49.69 ml and 64.74 ml, respectively, in the placebo vs. treatment arm. The volume increase was seen in both normal delivery and the LSCS groups. In the LSCS group, time to breast fullness was 40.65 hours and 32.2 hours, respectively, in the placebo vs. treatment arm and milk volumes were 52.35 ml and 66.67 ml, respectively, in the placebo vs. treatment arm. In the full-term normal delivery (FTND) group, time to breast fullness was 28.19 hours and 24.78 hours, respectively, in the placebo vs. treatment arm and milk volumes were 39.38 ml and 58.33 ml, respectively (Table 2).

**Table 2:** Time to breast fullness and milk volume extracted after 72 hours after delivery FTND - full-term normal delivery; LSCD - lower segment Caesarean section

Breast fullness and milk volume	Control group (placebo)			Study group (Shavari Bar®)			t-test
	N	Mean	SD	N	Mean	SD	p
LSCS delivery (n=61)							
Time to breast fullness (hours)	31	40.65	17.08	30	32.2	12.55	0.032
Total milk volume (ml)	31	52.35	24.72	30	66.67	25.2	0.029
FTND (n=17)							
Time to breast fullness (hours)	8	28.19	11.08	9	24.78	8.06	0.475
Total milk volume (ml)	8	39.38	27.44	9	58.33	13.23	0.084
All patients (n=78)							
Time to breast fullness (hours)	39	38.09	16.7	39	30.49	12	0.024
Total milk volume (ml)	39	49.69	25.48	39	64.74	23.11	0.008

**Figure 2:**
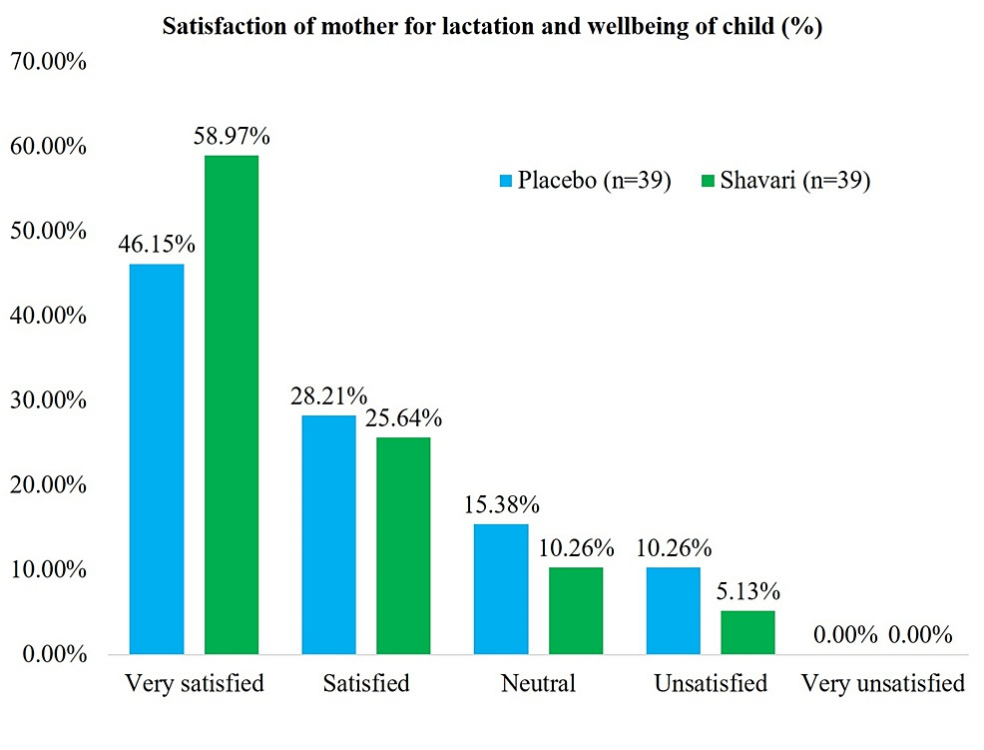
Satisfaction of mother with lactation and well being

In the global assessment of the satisfaction of mothers with lactation and the well-being of children in the treatment arm, 85% of mothers were satisfied to very satisfied, and in the placebo arm, this was 74% (Figure 2).

**Figure 3:**
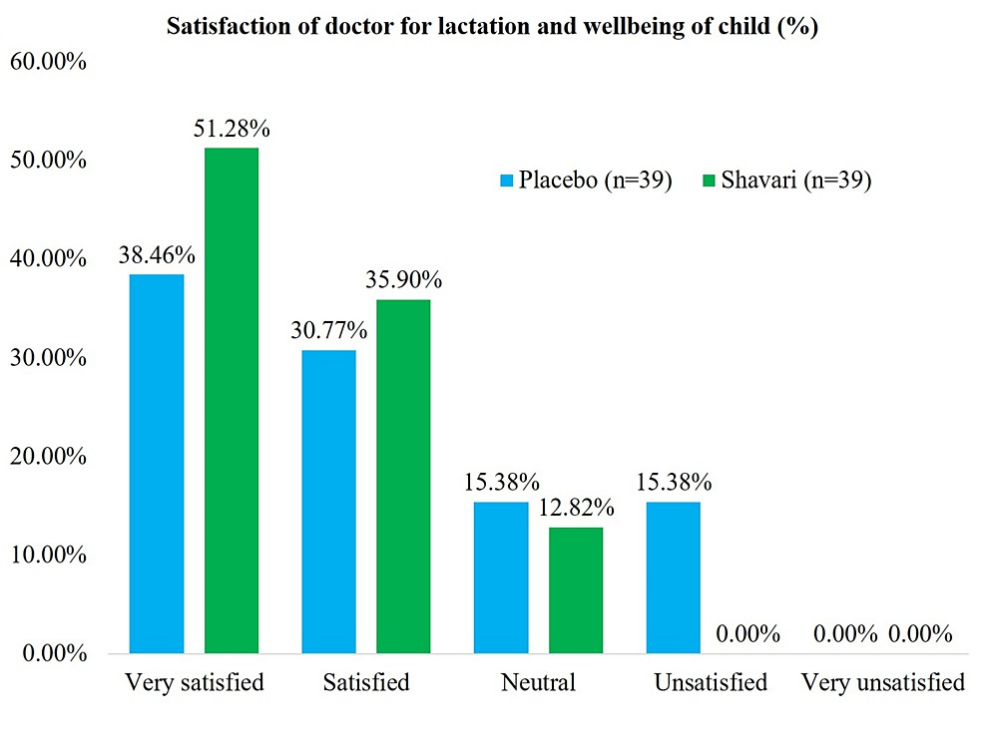
Satisfaction of doctor with lactation and well being

Similarly, in the global assessment of the satisfaction of doctors with lactation and well-being in the treatment arm, 87% of doctors were satisfied to very satisfied, and in the placebo arm, this was 69% (Figure 3).

**Figure 4:**
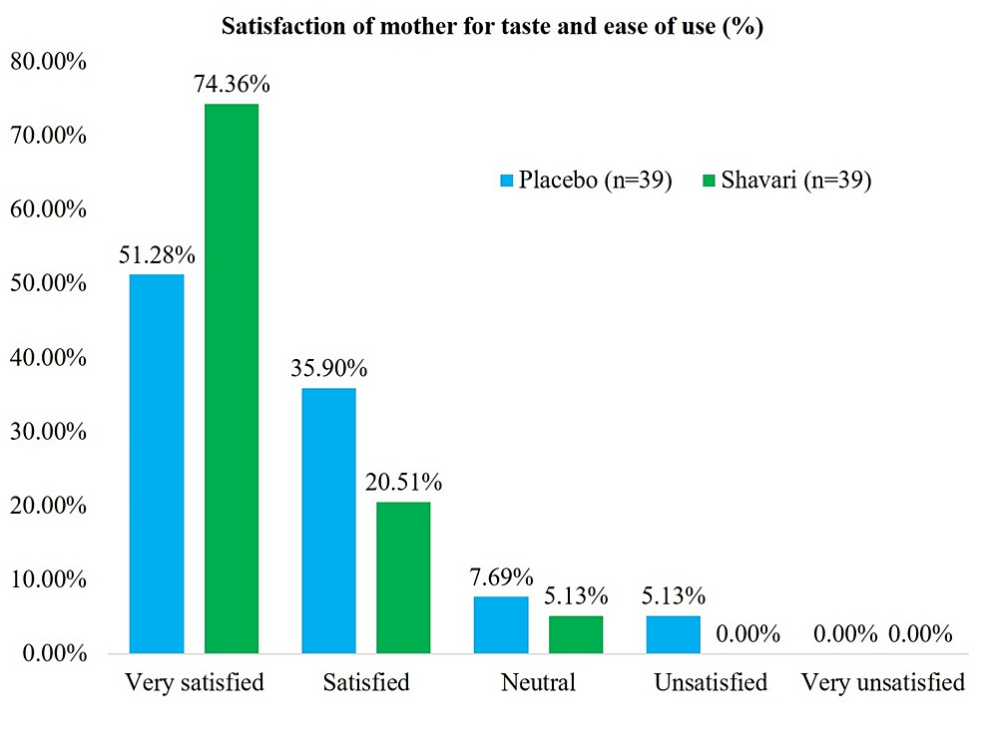
Satisfaction of mother with taste and ease of use

Satisfaction of mothers with taste and ease of use in the treatment arm, 95% of mothers were satisfied to very satisfied, and in the placebo arm, this was 72% (Figure 4).

 No adverse events were reported.

## Discussion

Many women face the concern of insufficient milk production, which leads to supplementation and early termination of breastfeeding. Non-pharmacological treatment in such women needs consideration for the influence of various maternal and neonatal factors like the baby's sucking and latching with the breast, and feeding frequency on milk production. Oral galactagogues are substances that stimulate milk production and are frequently used for improving breast milk output.

Breast milk provides the ideal nutrition for the infant, and WHO recommends exclusive breastfeeding for the first six months. Many women express concern about their ability to produce enough milk, and insufficient milk is frequently cited as the reason for supplementation and early termination of breastfeeding [2]. A longitudinal observational study that enrolled mothers who initiated breastfeeding after delivering healthy-term infants found that the rate of discontinuation of breastfeeding was 37% by the second week [3]. Mothers suffer from a feeling of failure and inferiority when they cannot produce sufficient milk for their babies. Despite all the knowledge and guidelines, it is still a common problem, and top feeds are given early postpartum due to insufficient breast milk production.

There is a high frequency of inadequate milk production in early lactation, which leads to the introduction of water or top feeds in the first few days after delivery. If top feeds are given instead of breastfeeds, it could have a negative impact on breast milk production. Milk supply in the first postpartum week is of critical importance. In the current study, two important parameters were accessed. The time to breast fullness was reduced in the treatment arm, and the quantity of milk produced increased. Early achievement of breast milk production can have a positive impact on the confidence of the mother and lead to continued efforts for breastfeeding.

Health care providers rely on non-pharmacological interventions and usually are in a dilemma as many galactagogues are not backed up with robust evidence. Given the suboptimal rates of exclusive breastfeeding and the availability and demand for medical and herbal lactation therapies, controlled trials and analyses investigating these medicines are urgently warranted [7]. So, we conducted this randomized, double-blind clinical study on the Shavari bar.

Our findings indicate that the Shavari bar increases breast milk production in nursing mothers more effectively than a placebo. A statistically significant increase in breast milk volume was observed in the study arm. It is worth noting that most of the subjects enrolled had undergone LSCS. Mothers undergoing LSCS are less prone to breastfeeding and tend to delay breastfeeding initiation [8]. This was also observed in placebo arms of the study, where there was significant variation in time to breast fullness among LSCS and vaginal delivery placebo subgroups. The study arm showed a significant increase in milk volume. Importantly, no maternal or neonatal adverse events were observed in the study. The use of pharmacological galactagogues has been associated with side effects.

Also, this study found that Shavari bars reduce time to breast fullness and can help establish lactation early, which can avoid the introduction of water and top feeds and benefit the mother and neonate. Another strength of the current study was that it was a randomized placebo-controlled study regarded as higher quality evidence. The measurement of milk for most patients was taken in a hospital setting using a standard breast pump, so data variability was less, and variation due to differences in the method of milk extraction was less.

In the current study, the treatment with Shavari bars resulted in a statistically significant and clinically meaningful increase in milk production. This was in line with an earlier randomized, double-blind clinical study of Shatavari. The galactagogue effect of Shatavari was evaluated in 60 mothers by recording changes in their prolactin hormone levels [9]. The Shatavari group showed a more than three-fold increase in the prolactin hormone level compared to the control group. There was a substantial increase in the weight of the babies in the study group. Subjective satisfaction of the mother regarding the state of lactation and the well-being and happiness of the child was many folds more in the Shatavari group.

Compliance is again a very important aspect. Shatavari is available in various oral dosage forms like powder, granules, capsules, etc. However, many of these formulations have a very high content of sugar to mask the bitter taste of Shatavari. Also, most of these products have to be mixed with milk, so the palatability and taste are of concern. Shavari bar, the formulation that has been tested in the current study, is a granola bar having Shatavari and oats along with dry fruits, honey, and sweetened cocoa. It is natural and preservative-free. The study data showed that most of the patients gave satisfactory feedback about the taste. The global feedback in the treatment arm was better than in the placebo arm.

Our study had certain drawbacks. The sample size was small. The time to breast fullness was not found to be statistically significant among women with FTND in Shavari arm vs. placebo arm, possibly due to the smaller sample size. There was a statistically significant increase in the amount of milk produced in Shavari arm vs. the placebo arm (p<0.01); however, the same was not seen in the subgroup analysis, also possibly due to the smaller sample size. The data on Shatavari needs to be generated in a bigger sample size. Also, there was no measurement of the blood level of prolactin to correlate with the clinical findings. 

## Conclusions

The most crucial period for success or failure of breastfeeding is the first ten days after delivery, which is also the most stressful period for mothers. Perceived milk insufficiency is the major impediment as it leads to true milk insufficiency. It leads to a vicious cycle of top feeds, decreased sucking, and low milk production. Breastfeeding is almost universally started but is stopped very soon due to this myth; therefore, it is necessary to have various interventions to support breastfeeding in the initial few days postpartum. The current study supports the consideration of using the Shavari Bar® as an effective option to support women in the first few days of the postpartum period to establish lactation and build confidence in breastfeeding along with nonpharmacological intervention.

## References

[1] (2022). WHO: breastfeeding. https://www.who.int/health-topics/breastfeeding#tab=tab_1.

[2] Foong SC, Tan ML, Foong WC, Marasco LA, Ho JJ, Ong JH (2020). Oral galactagogues (natural therapies or drugs) for increasing breast milk production in mothers of non-hospitalised term infants. Cochrane Database Syst Rev.

[3] Ertem IO, Votto N, Leventhal JM (2001). The timing and predictors of the early termination of breastfeeding. Pediatrics.

[4] Kent JC, Gardner H, Geddes DT (2016). Breastmilk production in the first 4 weeks after birth of term infants. Nutrients.

[5] Alok S, Jain SK, Verma A, Kumar M, Mahor A, Sabharwal M (2013). Plant profile, phytochemistry and pharmacology of Asparagus racemosus (Shatavari): a review. Asian Pac J Trop Dis.

[6] Hajela R (2015). Understand lactation and lactation failure: fight the curse of insufficient breast milk. Sch J Appl Med Sci.

[7] Bazzano AN, Hofer R, Thibeau S, Gillispie V, Jacobs M, Theall KP (2016). A review of herbal and pharmaceutical galactagogues for breast-feeding. Ochsner J.

[8] Hobbs AJ, Mannion CA, McDonald SW, Brockway M, Tough SC (2016). The impact of caesarean section on breastfeeding initiation, duration and difficulties in the first four months postpartum. BMC Pregnancy Childbirth.

[9] Gupta M, Shaw B (2011). A double-blind randomized clinical trial for evaluation of galactogogue activity of asparagus racemosus willd. Iran J Pharm Res.

